# CRISPR/Cas9-mediated endogenous protein tagging for RESOLFT super-resolution microscopy of living human cells

**DOI:** 10.1038/srep09592

**Published:** 2015-04-20

**Authors:** Michael Ratz, Ilaria Testa, Stefan W. Hell, Stefan Jakobs

**Affiliations:** 1 Max Planck Institute for Biophysical Chemistry, Department of NanoBiophotonics, Am Fassberg 11, 37077 Göttingen, Germany; 2 University of Göttingen Medical Faculty, Dept. of Neurology, Robert-Koch-Str. 40, 37075 Göttingen, Germany

## Abstract

Overexpression is a notorious concern in conventional and especially in super-resolution fluorescence light microscopy studies because it may cause numerous artifacts including ectopic sub-cellular localizations, erroneous formation of protein complexes, and others. Nonetheless, current live cell super-resolution microscopy studies generally rely on the overexpression of a host protein fused to a fluorescent protein. Here, we establish CRISPR/Cas9-mediated generation of heterozygous and homozygous human knockin cell lines expressing fluorescently tagged proteins from their respective native genomic loci at close to endogenous levels. We tagged three different proteins, exhibiting various localizations and expression levels, with the reversibly switchable fluorescent protein rsEGFP2. We demonstrate the benefit of endogenous expression levels compared to overexpression and show that typical overexpression-induced artefacts were avoided in genome-edited cells. Fluorescence activated cell sorting analysis revealed a narrow distribution of fusion protein expression levels in genome-edited cells, compared to a pronounced variability in transiently transfected cells. Using low light intensity RESOLFT (reversible saturable optical fluorescence transitions) nanoscopy we show sub-diffraction resolution imaging of living human knockin cells. Our strategy to generate human cell lines expressing fluorescent fusion proteins at endogenous levels for RESOLFT nanoscopy can be extended to other fluorescent tags and super-resolution approaches.

Currently, the majority of live cell microscopy studies rely on cells transiently overexpressing a host protein fused to a fluorescent protein (FP). However, there is substantial evidence that transiently or constitutively overexpressed proteins may cause a multitude of artifacts including mislocalizations and protein aggregation^1^, aberrant organelle morphology^2^,^3^, violated balanced gene dosage^4^, and others^5^. These overexpression induced problems are presumably even more articulate in studies using diffraction-unlimited super-resolution microscopy, or nanoscopy, which facilitates the visualization of protein localizations and dynamics on a length scale inaccessible by conventional light microscopy. Furthermore, quantification of absolute protein numbers relying on transient overexpression is complex because next to the FP-tagged proteins also non-tagged proteins are present^6^. Emerging genome-editing techniques which enable the expression of fusion proteins from their native genomic loci are expected to largely avoid these problems^7^. Still, to our knowledge, so far all live cell super-resolution microscopy studies of mammalian cells using fluorescent proteins relied on overexpressed proteins.

Most of these concepts, including the methods called STED, PALM, dSTORM, GSDIM, RESOLFT have been successfully implemented with various fluorescent proteins^8^. RESOLFT microscopy stands out by the fact that the light dose required for overcoming the diffraction barrier is by several orders of magnitude lower than in the other super-resolution methods^9^,^10^,^11^,^12^,^13^. The light intensities required for RESOLFT microscopy, which are comparable to those applied in confocal microscopy, are particularly appealing for imaging living cells, where phototoxicity is always a prime concern^14^. To overcome the diffraction barrier, RESOLFT microscopy relies on the use of reversibly switchable fluorescent proteins (RSFPs) that can be repeatedly switched between a fluorescent and a non-fluorescent state by irradiation with light of different wavelength^9^,^10^ (for details on RESOLFT see Refs. 11–13). The RSFP rsEGFP2 is very well suited for RESOLFT microscopy, because it exhibits very good expression properties in mammalian cells, fast photo-switching, and good photostability^11^.

Recently, the type II bacterial clustered, regularly interspaced, short palindromic repeats (CRISPR)-associated (Cas) system has been engineered into a powerful widely used genome editing tool (for review see Refs. 15, 16). Compared to other genome editing methods including transcription activator-like effector nucleases (TALENs) or zinc finger nucleases (ZFNs), the CRISPR/Cas9 system is rapid, simple and inexpensive^17^,^18^. The Cas9 nuclease can be targeted to induce a DNA double-strand break (DSB) at any genomic site defined by a 20-bp long guide RNA (gRNA) sequence complementary to the target site that contains an upstream NGG protospacer-adjacent motif (PAM). Utilizing the endogenous homology-directed repair (HDR) pathway, a transgene, e.g. rsEGFP2, flanked by appropriate homologous sequences can be inserted at the site of the DSB. Because the genomic regulatory sequences are unaltered in such knockin cells, this is expected to result in close to endogenous expression levels of the fusion protein.

To our knowledge, CRISPR/Cas9 has so far not been used for locus-specific fluorescent protein integration into human cells. Such knockin cell lines would be expected to alleviate most problems otherwise associated to plasmid-driven overexpression allowing quantitative studies of protein dynamics at native expression levels^7^.

In this study, we present a straightforward and robust approach for the generation of human knockin cells using the CRISPR/Cas9 system. We demonstrate live cell RESOLFT microscopy to image human knockin cells expressing rsEGFP2 fusion proteins of different abundances and localizations from their native chromosomal loci.

## Results

### CRISPR/Cas9-mediated knockin of rsEGFP2 at HMGA1, VIM and ZYX loci

**Figure 1:**
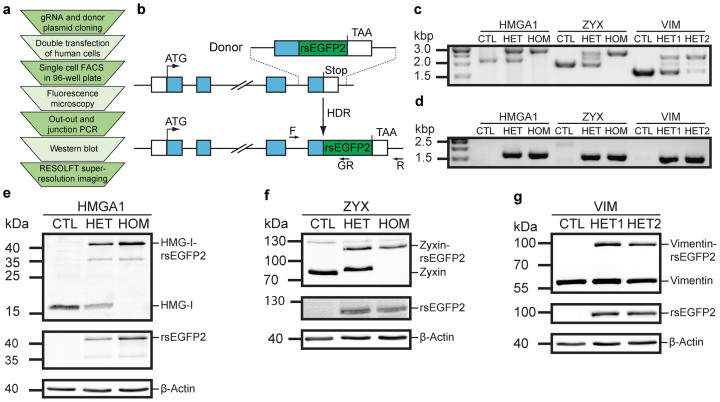
CRISPR/Cas9-mediated knockin of rsEGFP2 at three genomic loci in human U2OS cells. (a) Workflow for the generation of monoclonal human knockin cell lines for RESOLFT super-resolution microscopy. (b) Schematic representation of the integration strategy for generating C-terminally tagged rsEGFP2 fusion proteins expressed from the endogenous locus. White boxes, 5′- and 3′-untranslated region (UTR); blue boxes, exons; ATG, start codon; TAA, stop codon; HDR, homology-directed repair; F, locus-specific forward primer; R, locus-specific reverse primer; GR, rsEGFP2-specific reverse primer. (c, d) Analysis of two clonal lines per target locus. (c) Out-out PCR using primers F and R probing for locus-specific integration. CTL, control (parental U2OS cells); HMGA1-HET, heterozygous HMGA1-rsEGFP2^HET1.5^ clone, HMGA1-HOM, homozygous HMGA1-rsEGFP2^HOM2.4^ clone; ZYX-HET, heterozygous ZYX-rsEGFP2^HET^ clone; ZYX-HOM, homozygous ZYX-rsEGFP2^HOM^ clone; VIM-HET1, heterozygous VIM-rsEGFP2^HET1.2^ clone; VIM-HET2, heterozygous VIM-rsEGFP2^HET2.1^ clone. (d) Junction PCR using primers F and GR probing for locus-specific integration of rsEGFP2 transgene. (e–g) Western blot analysis of cell lysates of monoclonal cell lines immunoblotted for rsEGFP2, beta-Actin and the respective endogenously tagged protein: HMG-I (e), Zyxin (f) and Vimentin (g). Full length blots are shown in Supplementary Fig. 10.

We established a workflow (Fig. 1a) to create cell lines stably expressing C-terminal rsEGFP2 fusion proteins from the respective endogenous promotors using the CRISPR/Cas9 system (Fig. 1b). Specifically, we tagged the nuclear DNA-binding non-histone high mobility group protein HMG-I (gene: HMGA1), the class-III intermediate filament protein Vimentin (gene: VIM), and the focal adhesions plaque protein Zyxin (gene: ZYX). We choose these proteins for this study because they exhibit different expression levels and are localized in different cellular compartments, i.e. the nucleus (HMG-I), the cytoskeleton (Vimentin) and in the plasma membrane associated focal adhesion complexes (Zyxin). For each of the three target genes we designed two gRNAs (Supplementary Table 1) and one donor matrix (Supplementary Table 2) bearing homology arms of about 600 to 900 bp length to facilitate the integration of the rsEGFP2 coding sequence at the 3′-end of the respective last exon, replacing the stop codon but leaving the genomic locus otherwise unchanged. After co-transfection of human U2OS cells with a bicistronic plasmid encoding the gRNA and Cas9 together with the corresponding donor plasmid, single rsEGFP2-positive cells were sorted by fluorescence activated cell sorting (FACS) into 96-well plates. Of the six nuclease/donor matrix pairs tested, only gRNA2 targeting ZYX failed to result in cells expressing the expected fusion protein as judged by fluorescence microscopy prior to FACS. For all other gRNAs used, FACS-analysis revealed that between 0.1% and 4.7% of the transfected U2OS cells displayed a clearly discernible rsEGFP2 fluorescence signal (Supplementary Fig. 1). On average, between 10% and 20% of the sorted rsEGFP2 expressing single cells recovered and grew to confluency in the 96-well plate. This efficiency presumably could be further increased by supplementing the growth medium with antioxidants such as α-thioglycerol or bathocuprione disulphonate^19^,^20^.

Clones that expressed a fusion protein at the correct sub-cellular localization as determined by fluorescence microscopy (between 10% and 55% of the sorted clonal lines) were taken into culture and were analyzed by genomic PCR and Western blot for correct integration of the construct and expression of the fusion proteins (Fig. 1c–g and Supplementary Fig. 2). Per 96-well plate we identified between 2 and 9 knockin lines (Table 1). The nuclease/donor matrix pairs targeting the VIM locus resulted in 8 or 9 heterozygous monoclonal lines per 96-well plate, respectively, but no homozygous line. In case of ZYX-rsEGFP2 and HMGA1-rsEGFP2, we generated within each single 96-well plate one homozygous monoclonal knockin line. For further verification we sub-cloned out-out PCR fragments (using F and R primers, see Fig. 1b and Supplementary Table 3) for sequencing of the respective loci (Supplementary Figs. 3–7). We found for all targeted genes a base pair specific integration of the rsEGFP2 coding sequence without any sequence alterations within the coding regions. The ZYX-specific nuclease was targeted to the last exon for induction of a DSB. We avoided secondary nuclease-mediated mutations in the rsEGFP2 tagged ZYX allele by introducing silent mutations within the nuclease binding site in the donor plasmid. DNA sequencing revealed that non-homologous end joining induced in the non-tagged alleles of the heterozygous lines the deletion of a single cytosine in the last exon resulting in a frameshift that led to the expression of mutated non-tagged Zyxin (Supplementary Fig. 7c). In case of VIM and HMGA1, the respective nuclease targeted either the last intron (HMGA1-gRNA2) or the 3′ UTR (HMGA1-gRNA1, VIM-gRNA1 and 2). Consequently, we did not observe any alterations of the coding sequences, but found small indels (1 to 11 base pairs) in those non-coding regions (Supplementary Figs. 3–6). We conclude that CRISPR/Cas9-mediated genome editing combined with a hierarchical screening system is a powerful and straightforward approach to generate stable locus-specific human knockin cell lines.

### Expression level analysis in knockin cells and upon transient expression

**Figure 2:**
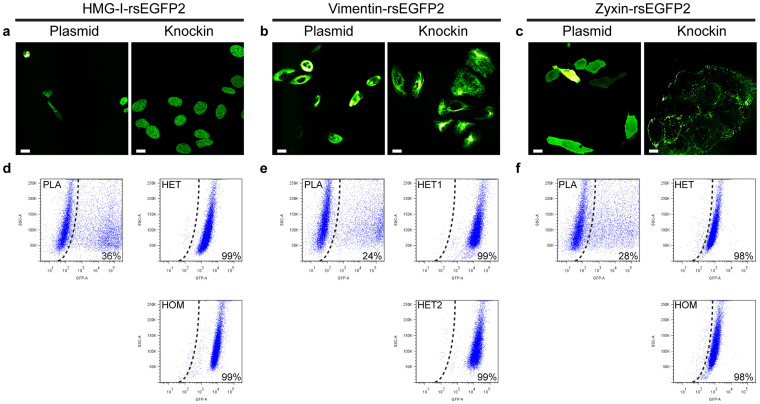
Expression level variability in knockin cells and upon transient expression. (a–c) Representative confocal images of U2OS cells expressing HMG-I-rsEGFP2 (a), Vimentin-rsEGFP2 (b) and Zyxin-rsEGFP2 (c) from a transiently transfected plasmid or from the respective native locus. (d–f) FACS analysis of U2OS cells expressing HMG-I-rsEGFP2 (d), Vimentin-rsEGFP2 (e) or Zyxin-rsEGFP2 (f) from a transiently transfected plasmid (PLA) or from the respective native locus (HET: heterozygous, HOM: homozygous). The black dotted line indicates the gate that separates non-transfected cells (left) from those with an rsEGFP2 signal (right; given in % of all analyzed cells). Non-transfected U2OS cells served as reference to set the gate. SSC-A, side-scatter area; GFP-A, Green Fluorescent Protein area. Scale bars: 20 μm.

FACS analysis of clonal knockin cell populations revealed very little variation in the expression levels of the rsEGFP2 fusion proteins (Fig. 2). A large portion of the plasmid transfected cells did not exhibit any rsEGFP2 fluorescence signal, whereas the remaining cells displayed a large variation of the fluorescence intensities (Fig. 2d–f). Whereas the intensity distributions of the fluorescent signals in case of the overexpressing cells seemed to be largely independent of the respective host protein, FACS revealed distinct expression levels for each knockin clone. In full accordance with the FACS data, microscopic inspection of individual genome-edited cell lines revealed little variation in the fluorescence signals (Fig. 2a–c) and the intracellular localizations of the fusion proteins were indistinguishable from the localizations of the endogenous proteins in wild type cells, as determined by immunofluorescence labelling (Supplementary Fig. 8). When the same fusion proteins were transiently expressed, we observed strongly varying expression levels, ectopic localizations, cell morphology changes as well as protein aggregations (Fig. 2a–c). We conclude that rsEGFP2 knockin cells are free of the artifacts observed in plasmid transfected cells.

### RESOLFT nanoscopy of rsEGFP2 knockin cells

**Figure 3:**
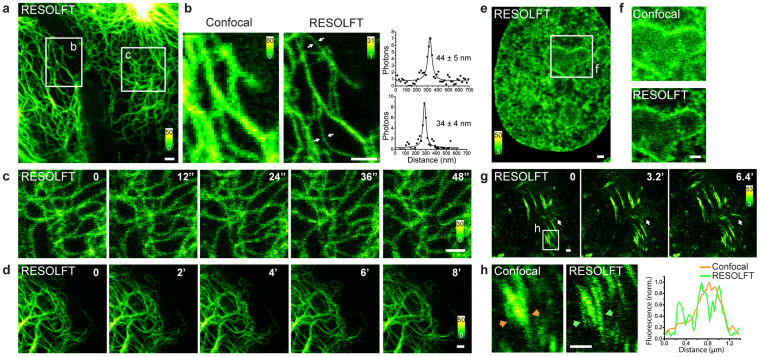
Live cell RESOLFT nanoscopy of CRISPR/Cas9 genome-edited rsEGFP2 knockin cells. (a–d) Heterozygous U2OS cells expressing Vimentin-rsEGFP2 from the endogenous locus. (a) Overview of two adjacent cells. (b) Comparison of confocal and RESOLFT images. The images have been recorded in the area indicated in (a). Right: Intensity profiles across the filaments at the indicated sites (white arrows), each fitted with a Lorentzian function. Given are the full width at half maximum (FWHM) values; for details see Supplementary Fig. 9. (c) Repeated RESOLFT imaging in the area indicated in (a). Images were recorded every 12 seconds, as indicated. (d) Time-lapse RESOLFT imaging; images were recorded every 2 minutes, as indicated. (e, f) RESOLFT microscopy of a homozygous cell expressing HMG-I-rsEGFP2. (e) Overview. (f) Comparison of confocal and RESOLFT images. The images have been recorded in the area indicated in (e). (g, h) RESOLFT microscopy of homozygous cells expressing Zyxin-rsEGFP2. (g) Images were recorded every 3.2 min., as indicated. (h) Comparison of confocal and RESOLFT images recorded in the area indicated in (g). Right: Intensity line profile across the area indicated in (h). All images display raw data. No deconvolution was applied. The RESOLFT image in (f) has been smoothed. Details on the imaging parameters are provided in the Supplementary Information. Scale bars: 1 μm.

RESOLFT live cell super-resolution microscopy^12^,^13^ was used to image knockin cells expressing Vimentin-rsEGFP2 (Fig. 3a–d), HMG-I-rsEGFP2 (Fig. 3e,f), or Zyxin-rsEGFP2 (Fig. 3g,h). Comparison with diffraction-limited confocal microscopy demonstrates the superior resolution provided by RESOLFT imaging (Figs. 3b, f, h). Without any image processing, we measured over stretches of thin Vimentin-rsEGFP2 filaments a full width at half maximum of ~40 nm, based on Lorentzian fits to the data (Fig. 3b and Supplementary Fig. 9). Sub-cellular dynamics were recorded both on the seconds time scale (Fig. 3c) as well as over the range of several minutes (Fig. 3d, g). If required, by alleviating the demand for the highest optical resolution, one could further decrease the imaging acquisition time and the light dose impinged on the sample. For example, doubling the pixel edge length would decrease the acquisition time by approximately a factor of four and reduce phototoxicity accordingly, providing additional flexibility for RESOLFT imaging. The recording speed could be further accelerated by using massively parallelized scanning^21^.

## Discussion

We demonstrate the use of CRISPR/Cas9 genome editing to generate heterozygous and homozygous human cell lines expressing rsEGFP2 fusion proteins. Whereas CRISPR/Cas9-mediated gene disruption can be highly efficient, base-pair specific integration by homologous repair following a Cas9 induced DSB is a relatively rare event. We found that single cell sorting by FACS allows a selection of positive clonal cell lines even at low integration efficiencies of less than 1%, rendering the generation of fluorescent protein knockin cell lines an accomplishable task even for non-specialized labs. The entire protocol, from the initial planning to the RESOLFT imaging of live clonal knockin cell lines, can be executed in less than six weeks. Obviously, this approach can easily be adopted to further fluorescent proteins or self-labeling enzymes such as the SNAP/CLIP/Halo-tag, and others.

So far, all super-resolution studies of live mammalian cells using fluorescent tags relied on the use of overexpressed proteins. Inevitably, overexpression is potentially associated to numerous artefacts, some of which may only be noticeable with the resolution provided by nanoscopy. FACS analysis and fluorescence microscopy revealed that the expression levels of the fusion proteins were largely constant within each clonal knockin cell line, whereas they varied by orders of magnitude in transfected cells, which likely accounts for many of the observed artefacts. Using the gene-edited cell lines and RESOLFT nanoscopy, we demonstrated an optical resolution of about 40 nm while allowing multiple recordings to visualize sub-cellular dynamics of HMG-I-, Vimentin- or Zyxin-rsEGFP2 fusion proteins expressed at endogenous levels in living cells. Instead of rsEGFP2, other suitable fluorescent tags could be used, opening up imaging at endogenous expression levels also to other super-resolution approaches, although not at the low light intensities provided by RESOLFT super-resolution microscopy. We propose that the combination of gene-editing and live cell super-resolution microscopy in human cells will be the method of choice for future quantitative analysis of endogenous protein numbers and localizations on the nanoscale.

## Methods

### Cell culture

U2OS cells (American Type Culture Collection, Manassas, VA, USA) were cultured in Dulbecco's modified Eagle's medium (DMEM) (Invitrogen, Carlsbad, CA, USA) supplemented with 10% fetal bovine serum (PAA, Pasching, Austria), 100 units/mL penicillin, 100 μg/mL streptomycin (all Biochrom, Berlin, Germany), and 1 mM sodium pyruvate (Sigma, St. Louis, MO, USA) under constant conditions at 37°C and 5% CO_2_.

### Nuclease plasmids

Design of the guide RNAs was carried out using the CRISPR Design Tool (http://crispr.mit.edu^22^) to minimize potential off-target effects. Oligonucleotide pairs (Supplementary Table 1) were cloned into the vector pX330^17^ as previously described in detail in Ref. 23. The final bicistronic vector encoded the gRNA and the Cas9 nuclease.

### Donor plasmids

DNA sequences for left homology arm (LHA) and right homology arm (RHA) were amplified from genomic DNA using the primer pairs listed in Supplementary Table 2. The length of the amplified homology arms was between 590 bp and 924 bp. In order to generate the Zyxin-rsEGFP2 donor plasmid, we introduced silent mutations within the Cas9 nuclease binding region of the left homology arm. To this end, a gBlock gene fragment containing the sequence was synthesized (Integrated DNA Technologies, Coralville, IA, USA). The coding sequence of rsEGFP2 was PCR amplified using the primers listed in Supplementary Table 2. For the VIM-rsEGFP2 donor plasmid, PCR products were purified, digested with KpnI/NotI (LHA), NotI/NcoI (rsEGFP2), NcoI/SalI (RHA) and cloned into a pUC57 plasmid (Fisher Scientific, Schwerte, Germany) that was digested with KpnI/SalI by a standard four fragments ligation. For HMGA1-rsEGFP2 and ZYX-rsEGFP2, the three PCR products (and the gBlock in case of ZYX-rsEGFP2 donor) were purified and cloned into a pUC57 plasmid that was digested with EcoRV using a one-step isothermal assembly reaction^24^.

### Transfection and clone isolation

U2OS cells were transfected with the bicistronic nuclease plasmids and the corresponding donor plasmids using FuGENE HD transfection reagent (Promega, Mannheim, Germany). To this end, 2 × 10^5^ cells per well were seeded in a 6-well plate with supplemented DMEM. The following day, transfection was carried out using a reagent to DNA ratio of 3.5 to 1 and a total DNA amount of 3 μg. Subsequently, the cells were further incubated at 37°C, 5% CO_2_. After seven days, cells were inspected by fluorescence microscopy. Wells containing cells exhibiting the expected sub-cellular localization of the rsEGFP2 fusion protein were subjected to single cell sorting into 96-well plates using a FACSAria II (BD Biosciences, Heidelberg, Germany). Within about two to three weeks after single-cell sorting, cells were split and transferred into 12-well plates containing glass cover slips. Cells expressing the fusion protein and showing the expected sub-cellular localization were identified using an epifluorescence microsope (DM6000B, Leica Microsystems, Wetzlar, Germany) equipped with an oil immersion objective (1.4 NA; 100×; Planapo; Leica) and a GFP filter cube (excitation filter: BP 470/40; emission filter: BP 525/50 nm) and were expanded for further experiments.

### Out-out PCR and junction PCR analysis for detection of targeted integration

U2OS clonal lines that showed a specific fluorescence signal for the respective structure were further analyzed using PCR. Genomic DNA was isolated using the DNeasy Blood & Tissue Kit (QIAGEN, Hilden, Germany) from a confluent well of a 12-well plate. 100 ng genomic DNA was used as a template for an out-out PCR (primers F and R, see Fig. 1b) or a junction PCR (primers F and GR, see Fig. 1b) analysis with the primers listed in Supplementary Table 3. PCR products were analyzed on a 1.5% agarose gel. Out-out PCR products of selected clonal lines were cloned into a pCR-Blunt II-TOPO vector (Invitrogen). The inserts were sequenced.

### Overexpression plasmids

Cloning of overexpression constructs was carried out using the primers listed in Table Supplementary Table 4. rsEGFP2 DNA sequences were amplified by PCR. VIM DNA was amplified from the plasmid pmKate2-vimentin (Evrogen, Moscow, Russia). HMGA1 DNA was amplified from pDONR223-HMGA1 (Human ORFeome, Internal ID: 4996). ZYX DNA was amplified from pDONR223-Zyxin (Human ORFeome, Internal ID: 4546). The respective PCR products were purified and used for one-step isothermal assembly^24^ with EcoRV-digested pFLAG-CMV-5.1 (Sigma Aldrich). In this plasmid, the fusion protein expression was driven by a CMV promoter. The peptide linkers between the rsEGFP2 and the respective host protein were identical in plasmid based expression and native expression.

For transfection using FuGENE HD transfection reagent (Promega), 2 × 10^5^ cells per well were seeded in a 6-well plate with supplemented DMEM. The following day, transfection was carried out using a reagent to DNA ratio of 3.5 to 1 and a total DNA amount of 3 μg. Images were recorded 1 to 3 days after transfection.

### Western blotting

For lysate preparation, cells (one confluent well of a 6-well plate) were washed two times in cold phosphate-buffered saline (PBS). Cells were scraped from the growth surface and resuspended in cold radioimmunoprecipitation assay (RIPA) buffer containing EDTA and complete protease inhibitor cocktail (Roche, Basel, Switzerland). After 20 min incubation on ice, the suspension was centrifuged at 13,000 rpm at 4°C for 30 min. The supernatant was removed and the protein concentration was measured using the Bradford dye-binding method (Bio-Rad, CA, USA). Samples were separated by 10% or 15% SDS-PAGE and transferred to a nitrocellulose membrane (GE Healthcare, Freiburg, Germany) in transfer buffer (25 mM Tris, 190 mM glycine, 20% methanol) over night. The membrane was rinsed in Tris-buffered saline (TBS) with 0.1% Tween 20 (TBST) and incubated in 5% blocking buffer (5 g skim milk per 100 ml TBST) at room temperature for 1 h. Primary antibodies were diluted in blocking buffer and incubated with the membrane at room temperature for 1 h. The following primary antibodies were used: anti-HMGA1 (EPR7839; 1:5000; Abcam, Cambridge, UK), anti-Vimentin (V9; 1:1000; Santa Cruz Biotechnology, Heidelberg, Germany), anti-Zyxin (ZOL301, 1:1000, Abcam), anti-Actin (AC74; 1:3000, Sigma-Aldrich), anti-GFP (JL-8; 1:3000, Clontech, Saint-Germain-en-Laye, France). After washing with TBST the membranes were incubated at room temperature for 1 h with HRP-conjugated anti-rabbit or anti-mouse secondary antibodies (Dianova, Hamburg, Germany) diluted 1:5000 in blocking buffer. After washing with TBST the membrane was incubated with Pierce ECL western blotting substrate (Fisher Scientific) and exposed to a CCD camera. Membranes were stripped using mild stripping buffer (15 g Glycine, 0.001% SDS, 0.01% Tween 20, pH 2.2) followed by the described protocol for reprobing with a different antibody.

### Immunostaining and confocal imaging

U2OS cells were grown on glass coverslips in 6-well plates overnight. Vimentin staining was carried out on cold methanol (−20°C) fixed cells. For Zyxin or HMG-I staining, cells were fixed in 4% formaldehyde. After cell fixation, coverslips were incubated in 2% blocking buffer (2 g BSA per 100 ml PBS). Primary antibodies were diluted in blocking buffer and incubated with the coverslips at room temperature for 1 h. The following primary antibodies were used: anti-HMG-I (EPR7839; 1:400; Abcam), anti-Vimentin (V9; 1:100; Santa Cruz Biotechnology), anti-Zyxin (ZOL301, 1:400, Abcam). After washing in blocking buffer, KK114-coupled secondary antibodies^25^ were diluted 1:50 and added for incubation at room temperature for 1 h. After three PBS washing steps, cells were mounted in Mowiol for imaging. Cells were visualized with a confocal microscope (TCS SP5, Leica) equipped with an oil objective (HCX PL APO CS 63× oil immersion objective) and a 633 nm HeNe continuous wave laser. Each image was averaged at least twice.

### RESOLFT microscope

The home-built RESOLFT microscope utilized three separate beam paths for generating co-aligned focal spots: two at a wavelength of 491 nm for excitation and OFF-switching, and one at 405 nm for ON-switching. The two focal spots at 491 nm comprised: (i) a normally focused pulsed beam for reading out the fluorescence signal; (ii) a ‘doughnut-shaped’ focal intensity distribution with a central minimum (‘zero’) for OFF-switching at the focal periphery in the xy-plane, obtained by passing a continuous wave beam through a vortex phase mask (463 nm mask, vortex plate VPP-A, RPC Photonics, Rochester, NY). The two focal intensity spots were generated by two different lasers diodes: one for OFF-switching (50 mW, continuous wave, Calypso 50, Cobolt, Stockholm, Sweden) and the second (10 mW, 80–100 ps pulse width PicoQuant, Berlin, Germany) for fluorescence readout. The third focal spot, again with a regularly focused profile, was generated by a laser diode at 405 nm wavelength (30 mW, BCL-030-405-S, CrystaLaser, Reno, NV, USA) and used for the ON-switching of the fluorescent protein. An oil-immersion objective lens (HCX PC APO, 100×, 1.4 NA, oil; Leica Microsystems, Wetzlar, Germany) was used to image the different cell lines. A piezo actuator (ENV40/20, Piezosystem Jena, Jena, Germany) was used to move the objective lens along the optical axis in a range of 120 μm. A separate piezo stage (NV40, Piezosystem Jena) was implemented to translate the sample with nanometer precision in the xy-plane. The fluorescence signal was filtered by a bandpass filter (532/70 nm) and detected by an epitaxial silicon single photon avalanche diode SPAD (MPD, Bolzano, Italy); fluorescence photons were counted only when the 491 nm pulse read-out beam was switched on. The individual laser beam paths were triggered either by an acousto-optic modulator (MTS 130A3, Pegasus Optik GmbH, Wallenhorst, Germany) or by an acousto-optic tunable filter (AOTF.nC/TN, Pegasus Optik GmbH). The pulse sequence and duration were defined by a pulse generator (Model 9514, QUANTUM COMPOSERS, Bozeman, MT, USA) and triggered by a time-correlated single photon counting module (Becker & Hickl, Berlin, Germany) pixel by pixel.

### RESOLFT imaging

Each image was recorded by applying a specific pulse scheme, pixel by pixel. For details on all shown images, see Supplementary Table 5. All intensity values are referring to the light intensities in the focal plane. Image acquisition was performed with the software ImSpector.

## Author Contributions

M.R. and S.J. designed experiments. M.R. and I.T. performed the experiments. M.R., I.T., S.W.H. and S.J. analyzed the data. M.R. and S.J. wrote the manuscript. All authors commented on the final manuscript.

## Supplementary Material

Supplementary Information Supplementary Information

## Figures and Tables

**Table 1:** Generated knockin cell lines. GFP+: Fraction of nuclease/donor pair transfected cells exhibiting rsEGFP2 fluorescence as determined by FACS analysis of 10,000 cells (see also Supplementary Figure 1). Single GFP+ cells were sorted into 96-well plates. The numbers refer to the analysis of clones obtained from a single 96-well plate

Cell line	GFP+ (%)	Genotyped	Homozygous	Heterozygous	No integration
**VIM-rsEGFP2^Endo1^**	0.77	11	-	8	3
**VIM-rsEGFP2 ^Endo2^**	0.73	10	-	9	1
**ZYX-rsEGFP2 ^Endo1^**	0.12	2	1	1	-
**ZYX-rsEGFP2 ^Endo2^**	0.06	-	-	-	-
**HMGA1-rsEGFP2 ^Endo1^**	4.72	6	1	5	-
**HMGA1-rsEGFP2 ^Endo2^**	2.23	6	1	4	1
