# Supplementary material for: CRISPR/Cas9-mediated endogenous protein tagging for RESOLFT super-resolution microscopy of living human cells
Source: Sci Rep. 2015 Apr 20;5:9592. doi: 10.1038/srep09592 (PMC4402611; doi:10.1038/srep09592)
Supplement: Supplementary Information [file srep09592-s1.pdf]

Supplementary Information

CRISPR/Cas9-mediated endogenous protein tagging for RESOLFT super-resolution microscopy of living human cells

Michael Ratz, Ilaria Testa, Stefan W. Hell, and Stefan Jakobs

Supplementary Table 1: Primers used for gRNA cloning into pX330 backbone.

Gene	gRNA name	Direction	Sequence (5’ to 3’)
**HMGA1**	HMGA1-gRNA1	F	CACCGAGGAGCAGGCGGCACGCAT
		R	AAACATGCGTGCCGCCTGCTCCTC
	HMGA1-gRNA2	F	CACCGCCAACAACTGCCCACCTCAC
		R	AAACGTGAGGTGGGCAGTTGTTGGC
**VIM**	VIM-gRNA1	F	CACCGCGCAAGATAGATTTGGAAT
		R	AAACATTCCAAATCTATCTTGCGC
	VIM-gRNA2	F	CACCGTCAGGAGCGCAAGATAGATT
		R	AAACAATCTATCTTGCGCTCCTGAC
**ZYX**	ZYX-gRNA1	F	CACCGACAGAGCACGTGACCGTCC
		R	AAACGGACGGTCACGTGCTCTGTC
	ZYX-gRNA2	F	CACCGTGTCATCTGCCTCAATCGAC
		R	AAACGTCGATTGAGGCAGATGACAC

Supplementary Table 2: Primers used for donor plasmid cloning based on pUC57 backbone.

Donor plasmid	Primer name	Sequence (5' to 3')
**pHMGA1-rsEGFP2**	HMGA1_LHA_F	TCTCGCGAATGCATCTAGATGATGTGACCCACCACACTGCACTGG
	HMGA1_LHA_R	ATCCGCTGCCCTGCTCCTCCTCCGAGGACTCCTGC
	rsEGFP2_HMGA1_F	GGAGGAGCAGGGCAGCGGATCCGGCGTGAGCAAGGGCGAGGAGC
	rsEGFP2_HMGA1_R	CACGCATGGGGGTACCTTACTTGTACAGCTCGTCCATG
	HMGA1_RHA_F	GTAAGGTACCCCCATGCGTGCCGCCTGCTCCTCAC
	HMGA1_RHA_R	AGGCCTCTGCAGTCGACGATGTGAGCTCCTTGTTATTGGTGCCCCATCTG
**pVIM-rsEGFP2**	VIM_LHA_F	ATATTAGGTACCCCTTAAACCACTTCCTAAAGCAGCTACATGAAACAGCTTCACTAGACTACC
	VIM_LHA_R	TATATTGCGGCCGCTTCAAGGTCATCGTGATGCTGAGAAGTTTCGTTGATAACC
	rsEGFP2_VIM_F	ATATTAGCGGCCGCCGGTAGTGGTTCAGGGGTGAGCAAGGGCGAGGAGCTGTTCACCG
	rsEGFP2_VIM_R	TTAATTCCATGGTTACTTGTACAGCTCGTCCATGCCGAGAGTGATCCC
	VIM_RHA_F	CGAAACTTCTCAGCATCACGATGACCTTCCATGGAAATTGCACACACTCAGTG
	VIM_RHA_R	TATTTAGTCGACGTTATGATTAAAATCACAGACCTGAGCTCATTTTAGAGAGACCTATCTTC
**pZYX-rsEGFP2**	ZYX_LHA_F	TCTCGCGAATGCATCTAGATGAAAGCCCTGGCTAACTCGGCTGGC
	ZYX_LHA_R	TACCCCACCCCATCCAGCTCCATCTTG
	rsEGFP2_ZYX_F	GGCAGCGGCACCGGTGTGAGCAAGGGCGAGGAGC
	rsEGFP2_ZYX_R	CTGTCCTCACGCTAGCTTACTTGTACAGCTCGTCCATG
	ZYX_RHA_F	GTAAGCTAGCGTGAGGACAGGCCCTCTTCAGACCG
	ZYX_RHA_R	AGGCCTCTGCAGTCGACGATGACGCAGATGGGAATCACACTGCCC
	ZYX_gBlock	GAGCTGGATGGGGTGGGGTAGGGTGGAGCAGAGCAGGGGCCTTCCGGTCCAGTGCCCCTCACCCTTCCTTCTTCCCAGGACTGCGGGAAGCCTCTGTCAATTGAGGCTGATGACAATGGCTGCTTCCCTCTGGAAGGTCATGTGCTCTGTCGGAAGTGCCACACTGCTAGAGCCCAGACCGGCAGCGGCACCGGTGTGAG

Note: 'ZYX gBlock' is a double-stranded DNA fragment and the given sequence corresponds to the 5' to 3' sequence of the plus strand.

Supplementary Table 3: Primers used for out-out and junction PCRs.

Target gene	Primer name	Sequence (5’ to 3’)
**HMGA1**	HMGA1_out_F	GTCCCTCCCTCTCCTGCTCCTAGAATACTCAG
	HMGA1_out_R	CAATGACGGATGTCGAAGAATGGAACATTGAAC
	GFP_R	CACGCTGCCGTCCTCGATGTTG
**VIM**	VIM_out_F	CTAATCTGGATTCACTCCCTCTGGTTGATACC
	VIM_out_R	CCTATATCTTGGCAACTTCCTATGCTTTAACTCCC
	GFP_R	CACGCTGCCGTCCTCGATGTTG
**ZYX**	ZYX_out_F	CTTGCTTTGGGAGAGTGACTGGTGAGGC
	ZYX_out_R	GCTGAGTAAGGAGCTGGAGCTGTCTGGGC
	GFP_R	CACGCTGCCGTCCTCGATGTTG

Supplementary Table 4: Primers used for one-step cloning of overexpression constructs based on pFLAG-CMV-5.1 backbone.

Plasmid name	Primer name	Sequence (5’ to 3’)
**pCMV-VIM-rsEGFP2**	VIM_CMV_F	TTCATCGATAGATCTGATGCCACCATGTCCACCAGGTCCGTGTC
	VIM_CMV_R	GAACCACTACCGGCGGCCGCTTCAAGGTCATCGTGATGCTGAG
	rsEGFP2_CMV_F	CCGCCGGTAGTGGTTCAGGGGTGAGCAAGGGCGAGGAGC
	rsEGFP2_CMV_R	GTCGACTGGTACCGATTTACTTGTACAGCTCGTCCATGCC
**pCMV-ZYX-rsEGFP2**	ZYX_CMV_F	TTCATCGATAGATCTGATGCCACCATGGCGGCCCCCCGCCCGTCTC
	ZYX_CMV_R	CCGGTGCCGCTGCCGGTCTGGGCTCTAGCAGTGTGGCACTTCCG
	rsEGFP2_CMV_F	CCGGCAGCGGCACCGGTGTGAGCAAGGGCGAGGAGCTG
	rsEGFP2_CMV_R	GTCGACTGGTACCGATTTACTTGTACAGCTCGTCCATGCCGAG
**pCMV-HMGA1-rsEGFP2**	HMGA1_CMV_F	TTCATCGATAGATCTGATGCCACCATGAGTGAGTCGAGCTCGAAGTCC
	HMGA1_CMV_R	CGGATCCGCTGCCCTGCTCCTCCTCCGAGGACT
	rsEGFP2_CMV_F	CAGGGCAGCGGATCCGGCGTGAGCAAGGGCGAGGAGCTG
	rsEGFP2_CMV_R	GTCGACTGGTACCGATGGTACCTTACTTGTACAGCTCGTCCATGCCGAG

Supplementary Table 5: Overview of parameters used for RESOLFT imaging of endogenously tagged cells.

**Cell line**	**VIM-rsEGFP2[HET1.2]**	**ZYX-rsEGFP2[HOM]**	**HMG-I-rsEGFP2[HOM2.4]**
**Pixel size (nm)**	20 (overview)<br>15 (inset and **Suppl. Fig. 9**)<br>30 (movie)	30	30 (x-axis)<br>25 (y-axis)
**Overall dwell time (µs)**	520	1000	780
**ON switching at 405nm**<br>**(Gaussian shaped)**<br>**Time/Average Power/ Intensities**	50 µs<br>0.8 µW<br>2 kW/cm²	730 µs<br>2.6 µW<br>2 kW/cm²	50 µs<br>0.8 µW<br>2 kW/cm²
**OFF switching at 491 nm**<br>**(donut shaped)**<br>**Time/Average Power/ Intensities**	400 µs<br>8.8 µW<br>6.5 kW/cm²	730 µs<br>2.6 µW<br>2 kW/cm²	650 µs<br>3 µW<br>2.2 kW/cm²
**Read out at 491 nm**<br>**(Gaussian shaped)**<br>**Time/Average Power/ Intensities**	50 µs<br>2.4 µW<br>5.3 kW/cm²	200 µs<br>4.8 µW<br>2.6 kW/cm²	50 µs<br>4 µW<br>2.2 kW/cm²

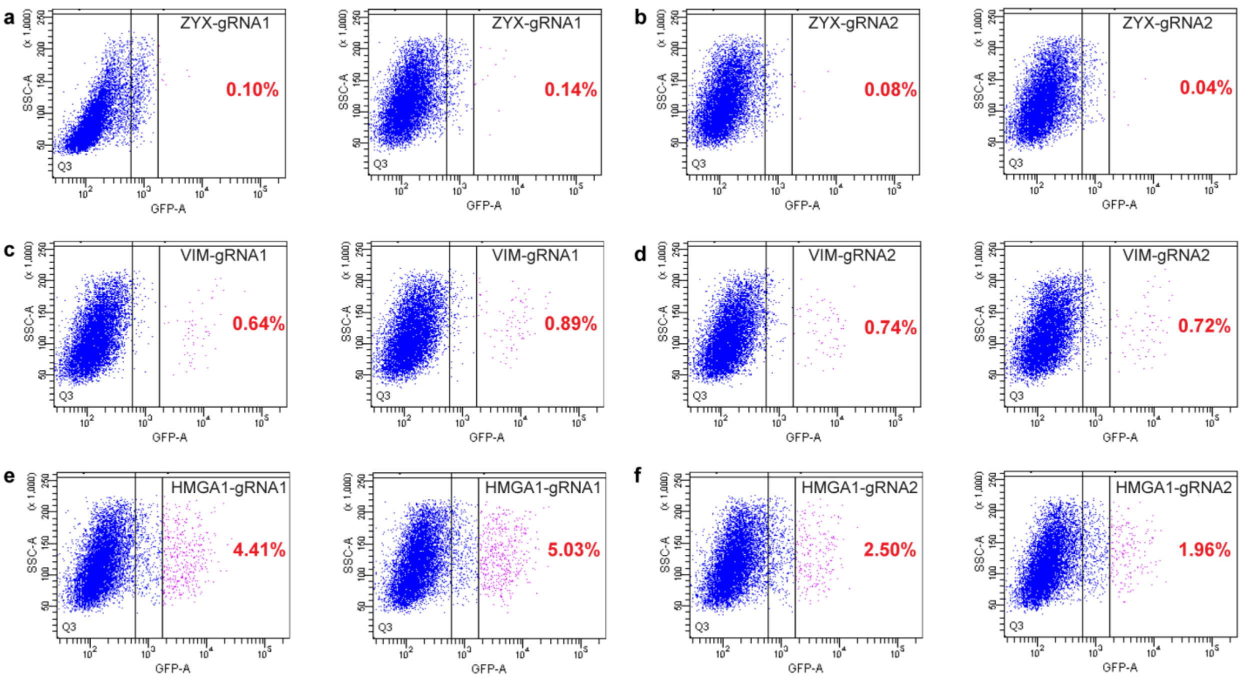

Supplementary Figure 1: Fluorescence activated cell sorting (FACS) dot plots. Cells were transfected with bicistronic Cas9/gRNA constructs and the respective donor plasmids. Seven days after transfection the amount of rsEGFP2 positive cells (given in %) was determined using FACS (threshold was chosen dependent on FACS sort of untransfected U2OS cells). For each experiment 10,000 cells were sorted. Dot plots of duplicate transfection experiments are shown. (**a, b**) Amount of rsEGFP2 positive cells for tagging of Zyxin using ZYX-gRNA1 (**a**) or ZYX-gRNA2 (**b**), respectively; (**c, d**) Amount of rsEGFP2 positive cells for tagging of Vimentin using VIM-gRNA1 (**c**) or VIM-gRNA2 (**d**), respectively; (**e, f**) Amount of rsEGFP2 positive cells for tagging of HMG-I using HMGA1-gRNA1 (**e**) or HMGA1-gRNA2 (**f**). SSC-A, side-scatter area; GFP-A, Green fluorescent protein area; blue dots, non-fluorescent cells; pink dots, GFP-positive cells.

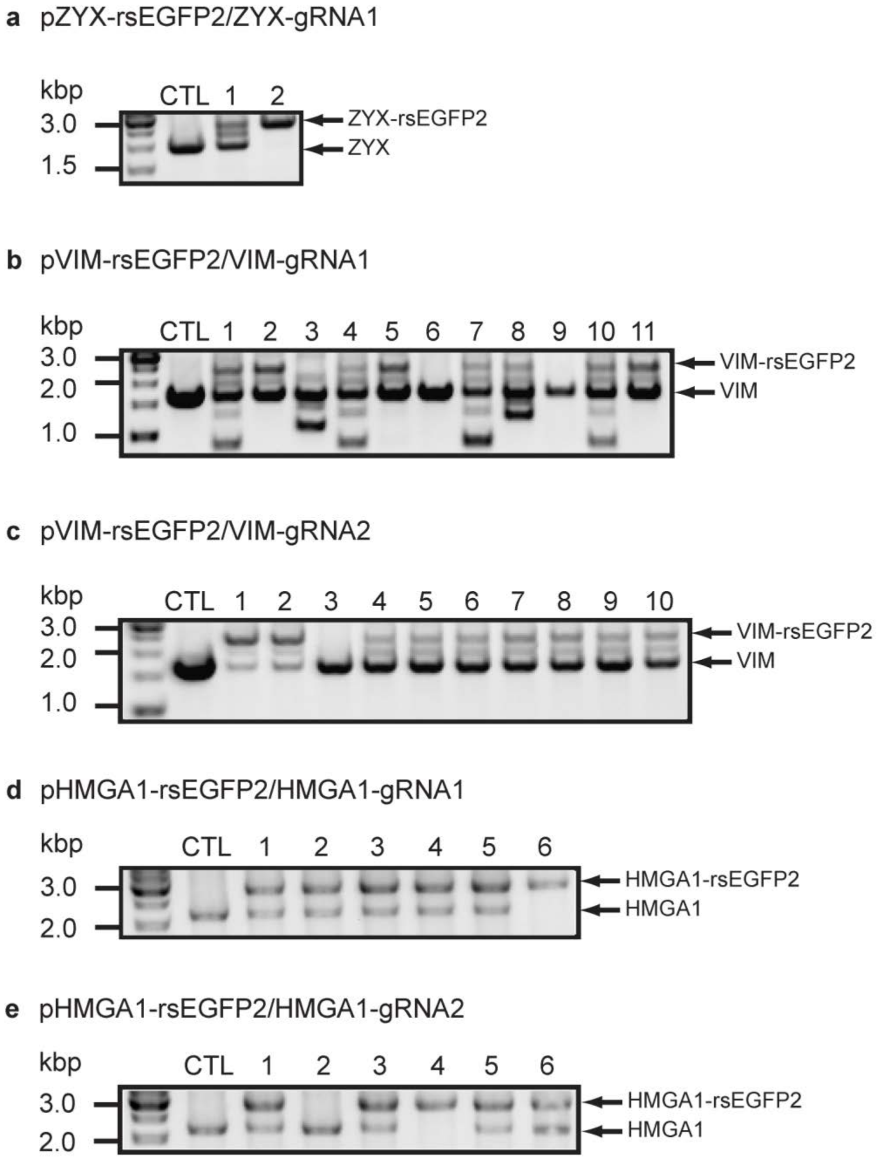

Supplementary Figure 2: out-out PCR analysis of monoclonal U2OS cell lines after FACS. Screening for site-specific integration was done using primers annealing outside of the homology arms of the donor plasmid (see Supplementary Table 3). (**a**) Analysis of ZYX-rsEGFP2 clones generated with ZYX-gRNA1. (**b, c**) Analysis of VIM-rsEGFP2 clones generated with VIM-gRNA1 (**b**) and VIM-rsEGFP2 clones generated with VIM-gRNA2 (**c**). (**d, e**) Analysis of HMGA1-rsEGFP2 clones generated with HMGA1-gRNA1 (**d**) and HMGA1-rsEGFP2 clones generated with HMGA1-gRNA2 (**e**).

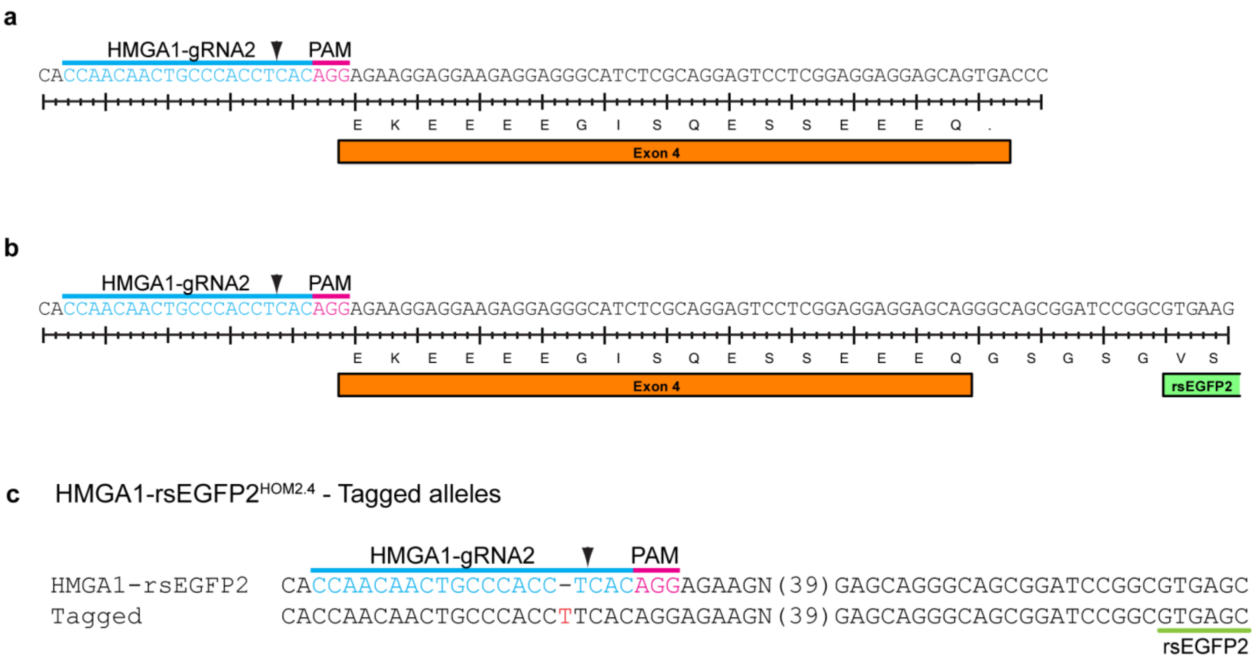

Supplementary Figure 3: Sequencing of clone HMGA1-rsEGFP2$^{HOM2.4}$ generated with HMGA1-gRNA2. (**a, b**) The gRNA2 cleavage site lies upstream of the HMGA1 stop codon within the non-coding region in both the untagged (**a**) and the tagged (**b**) allele. (**c**) Sanger sequencing of out-out PCR products for the tagged allele reveals a single base pair insertion within the Cas9 binding site. Blue letters, Cas9 binding site; Pink letters, Protospacer-adjacent motif (PAM); Black arrow, Cas9 cleavage site.

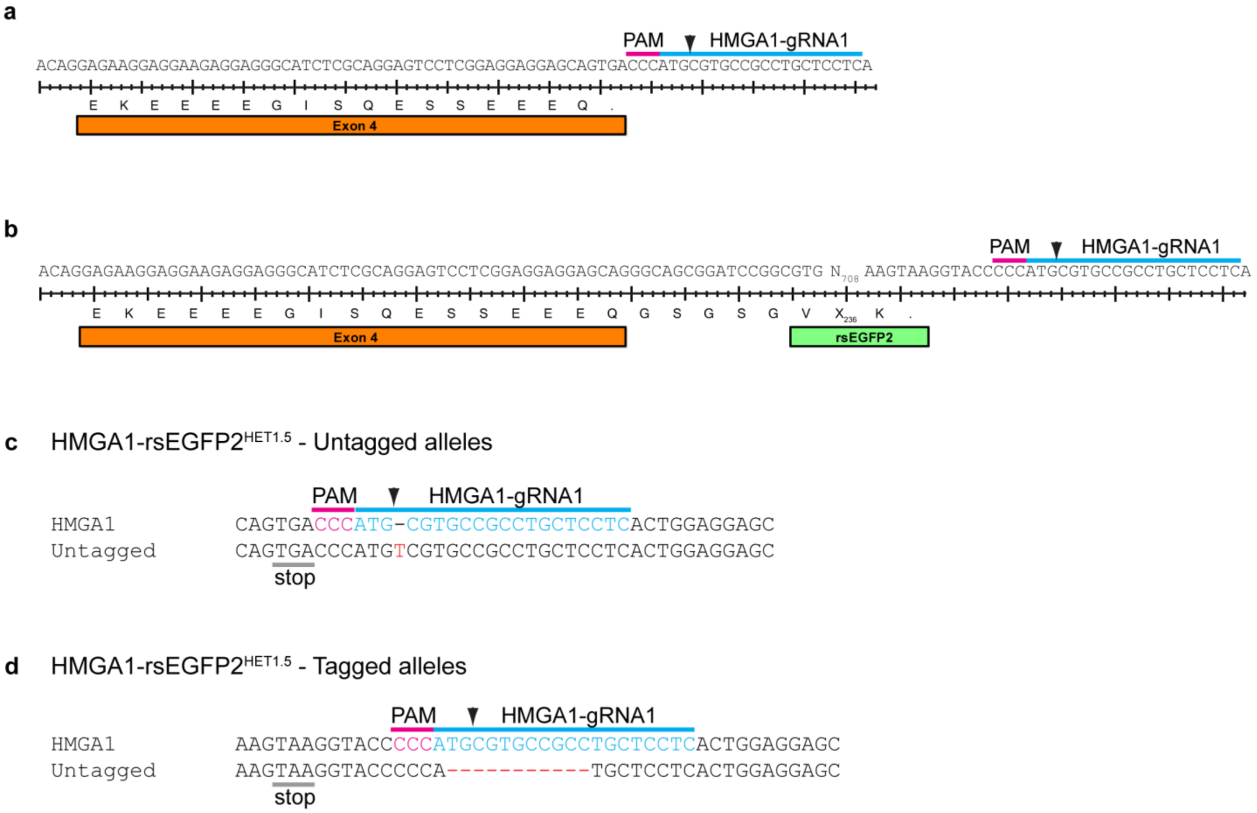

Supplementary Figure 4: Sequencing of clone HMGA1-rsEGFP2$^{HET1.5}$ generated with HMGA1-gRNA1. (**a, b**) The gRNA1 cleavage site lies downstream of the HMGA1 stop codon within the non-coding region in the untagged allele (**a**) and downstream of the rsEGFP2 stop codon in the tagged allele (**b**). (**c, d**) Sanger sequencing of out-out PCR products reveals a single base pair insertion within the Cas9 binding site for the untagged allele (**c**) and an 11 bp deletion for the tagged allele (**d**). Blue letters, Cas9 binding site; Pink letters, Protospacer-adjacent motif (PAM); Black arrow, Cas9 cleavage site.

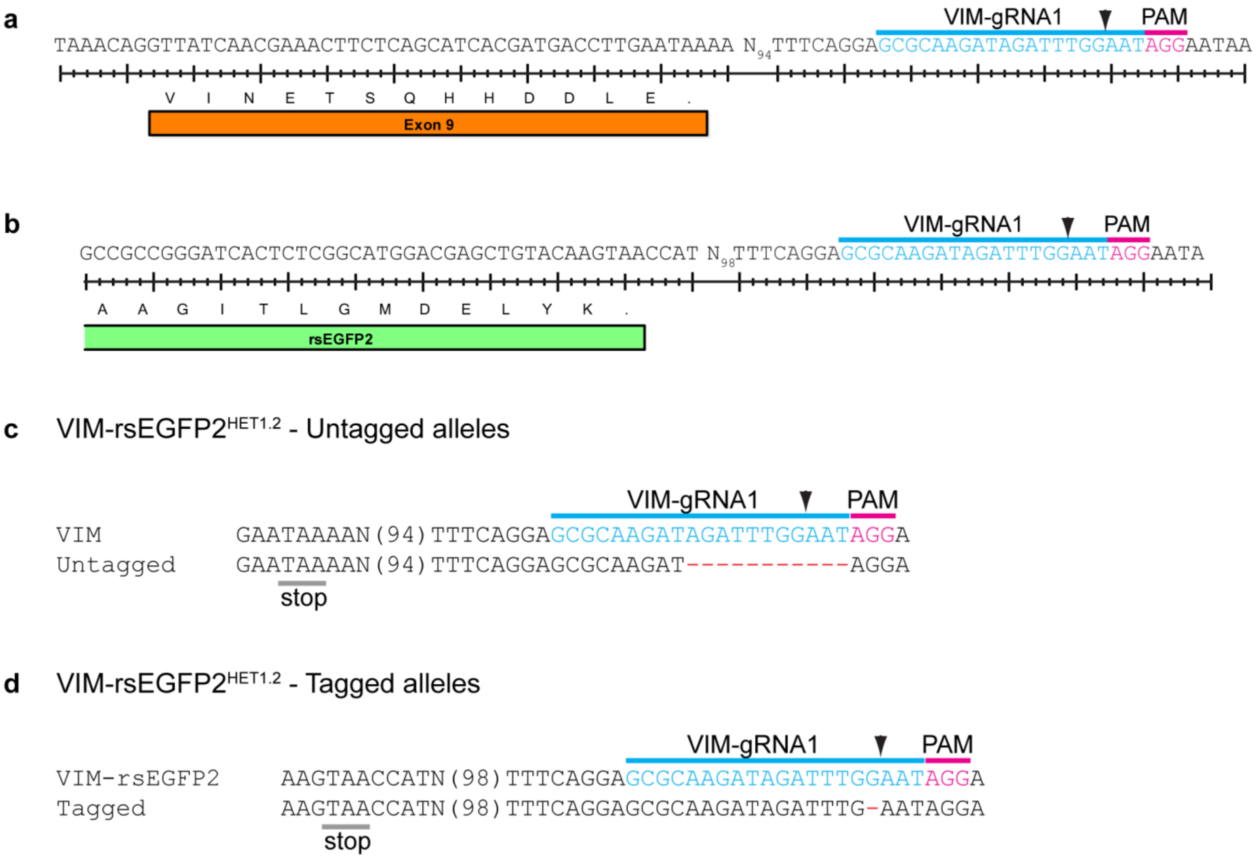

Supplementary Figure 5: Sequencing of clone VIM-rsEGFP2$^{HET1.2}$ generated with VIM-gRNA1. (**a, b**) The gRNA1 cleavage site lies downstream of the VIM stop codon within the non-coding region in the untagged allele (**a**) and downstream of the rsEGFP2 stop codon in the tagged allele (**b**). (**c, d**) Sanger sequencing of out-out PCR products reveals an 11 bp deletion within the Cas9 binding site for the untagged allele (**c**) and a single base pair deletion for the tagged allele (**d**). Blue letters, Cas9 binding site; Pink letters, Protospacer-adjacent motif (PAM); Black arrow, Cas9 cleavage site.

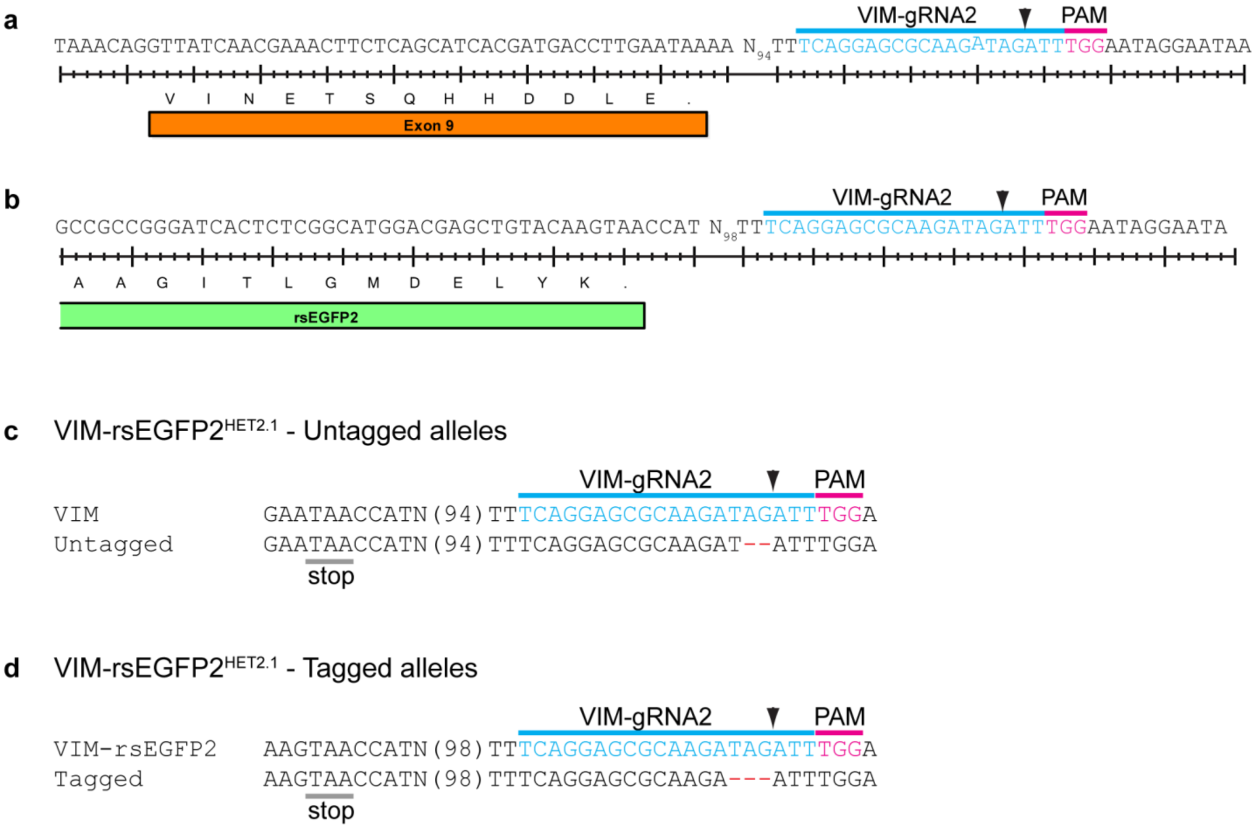

Supplementary Figure 6: Sequencing of clone VIM-rsEGFP2$^{HET2.1}$ generated with VIM-gRNA2. (**a, b**) The gRNA2 cleavage site lies downstream of the VIM stop codon within the non-coding region in the untagged allele (**a**) and downstream of the rsEGFP2 stop codon in the tagged allele (**b**). (**c, d**) Sanger sequencing of out-out PCR products reveals a 2 bp deletion within the Cas9 binding site for the untagged allele (**c**) and a 3 bp deletion for the tagged allele (**d**). Blue letters, Cas9 binding site; Pink letters, Protospacer-adjacent motif (PAM); Black arrow, Cas9 cleavage site.

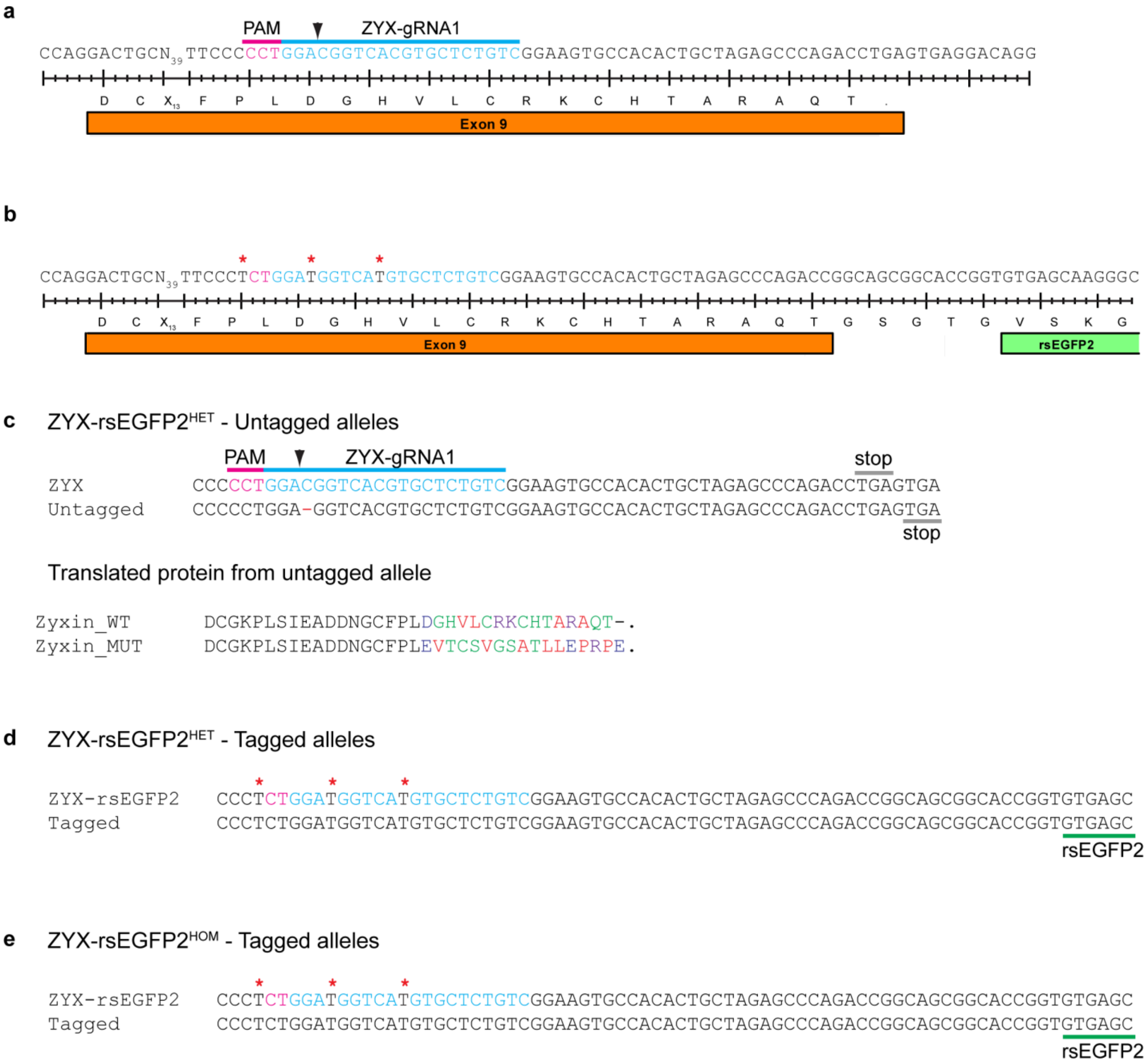

Supplementary Figure 7: Sequencing of clones ZYX-rsEGFP2^{HET} and ZYX-rsEGFP2^{HOM} generated with ZYX-gRNA1. (**a, b**) The gRNA1 cleavage site lies upstream of the ZYX stop codon within the last exon in the untagged allele (**a**) but will be blocked by introduction of silent mutations (red asterisks) in the tagged allele (**b**). (**c-e**) Sanger sequencing of out-out PCR products. A single base pair deletion within the Cas9 binding site for the untagged allele of the heterozygous clone resulted in a frameshift and the expression of mutant Zyxin (**c**). Cleavage of Cas9 in the tagged alleles was blocked by silent mutations in both the heterozygous (**d**) and the homozygous clone (**e**). Blue letters, Cas9 binding site; Pink letters, Protospacer-adjacent motif (PAM); Black arrow, Cas9 cleavage site.

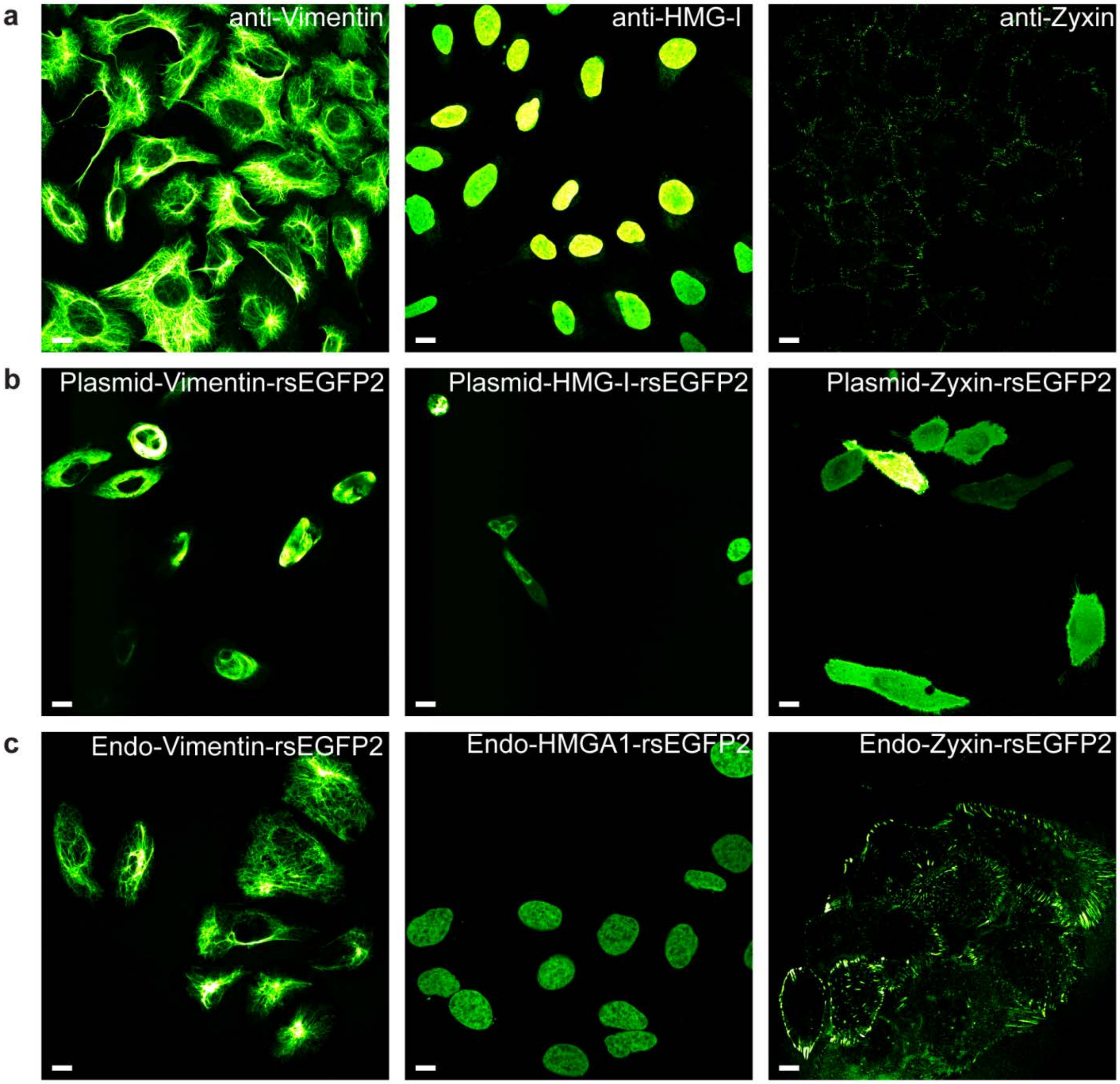

Supplementary Figure 8: Fluorescence confocal imaging of U2OS cells. (**a**) Fixed and immunostained U2OS cells show endogenous expression patterns of Vimentin, HMG-I and Zyxin. (**b**) Living and transfected U2OS cells show heterogenous expression levels of Vimentin-, HMG-I and Zyxin-rsEGFP2 fusion proteins under the control of a cytomegalovirus (CMV) promoter. (**c**) Living U2OS cells expressing Vimentin-, HMG-I- and Zyxin-rsEGFP2 fusion proteins from the endogenous locus show homogenous expression patterns comparable to immunostaining against the untagged protein in fixed cells. Scale bar, 10 µm.

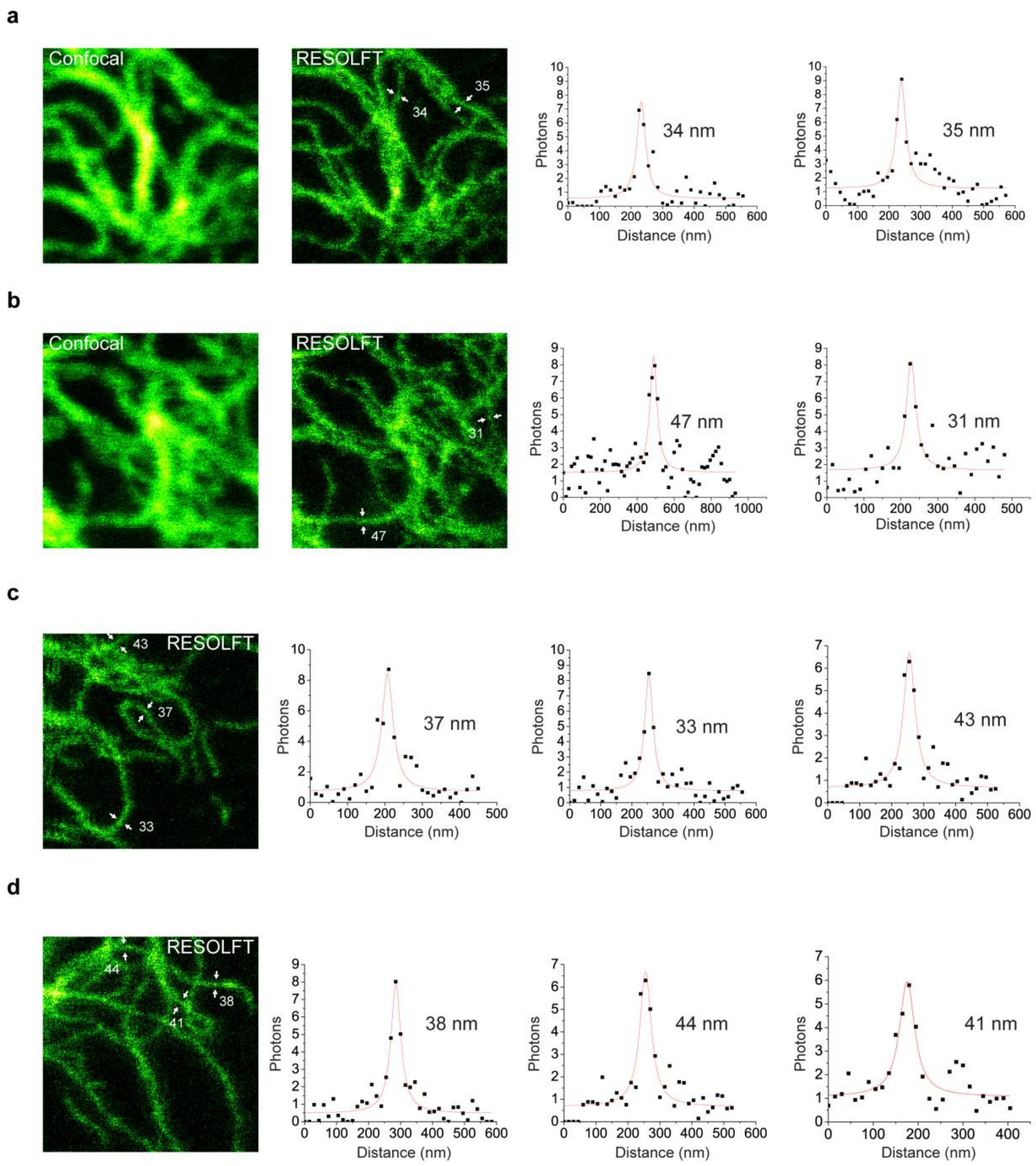

Supplementary Figure 9: Line Profiles. (**a-d**) Four different images (each is 3.8 x 3.8 µm with a pixel size of 15 nm) and selected line profiles within are shown. Each profile in the respective RESOLFT image was drawn over a filament (white arrows) using a line width of 2 pixels in ImageJ. The corresponding data points were exported to OriginPro 8.6, plotted and fitted with a Lorentzian peak function (red curve). The resolution was measured as the full width at half-maximum (FWHM) of the fitted curve. Using the given input values, the average resolution was 38 ± 5 nm.

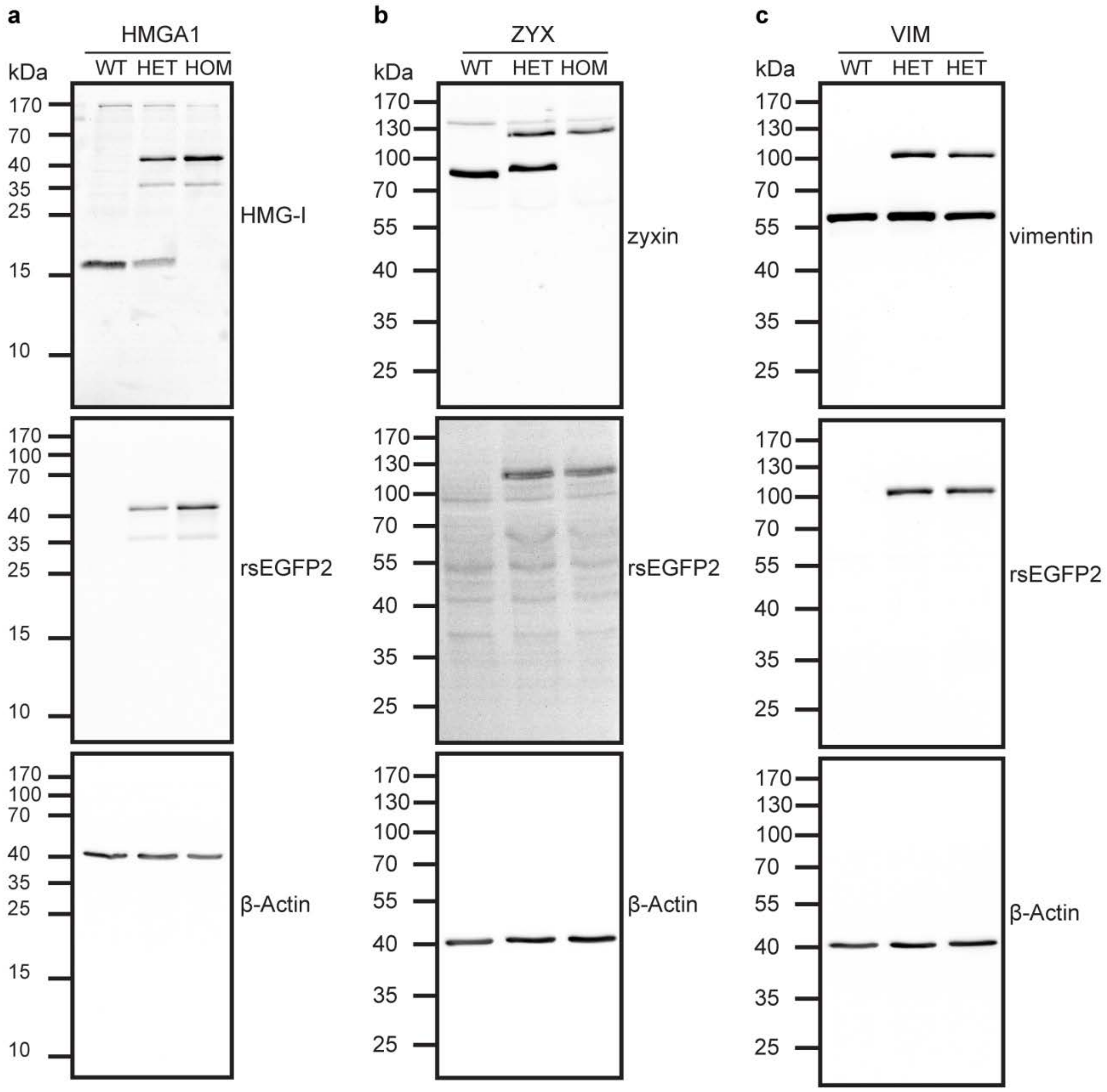

Supplementary Figure 10: Full length western blots. (**a-c**) Western blot analysis of cell lysates of monoclonal cell lines immunoblotted for GFP, actin and the respective endogenously tagged protein: HMG-I (**a**), Zyxin (**b**) and Vimentin (**c**). Full length blots of the cropped versions presented in Figure 1 are shown.
